# Clinical Characteristics and Outcomes of Neural Epidermal Growth Factor-like 1 Protein-Associated Membranous Nephropathy

**DOI:** 10.1016/j.ekir.2024.02.1405

**Published:** 2024-02-24

**Authors:** Suruthy Narayanasami, Niranjan Anitha Vijayakumar, Mayuri Trivedi, Aravind Sekar, Shabna Sulaiman, Saurabh Nayak, Neeraj Inamdar, Sourabh Sharma, Rajeevalochana Parthasarathy, Vidya Kadam, Vaibhav Keskar, Sahil Bagai, Ashwani Kumar, Bajinder Reen, Anila Abraham Kurien, Alok Sharma, Dinesh Khullar, Benil Hafeeq, Arvind Krishnakumar, Shafeeque Rahman KV, Ismail NA, Sanjeev Nair, Smita Divyaveer, Manish Rathi, Harbir Singh Kohli, Ritambhra Nada, Raja Ramachandran

**Affiliations:** 1 Department of Histopathology, Postgraduate Institute of Medical Education and Research, Chandigarh, India; 2 Department of Nephrology, Postgraduate Institute of Medical Education and Research, Chandigarh, India; 3 Division of Nephrology, LTMC, Sion, Mumbai, India; 4 IQRAA International Hospital and Research Centre, Kozhikode, India; 5 Department of Nephrology, All India Institute of Medical Sciences, Bathinda, India; 6 Dr. Hedgewar Hospital, Aurangabad, India; 7 Vardhman Mahavir Medical College, Delhi, India; 8 Madras Medical Mission, Chennai, India; 9 Lifeline Hospital, Thane and Lifeline hospital, LH Hiranandani Hospital, Mumbai, India; 10 Fortis Hospital, Mumbai, India; 11 Max Super Speciality Hospital, Saket, New Delhi, India; 12 William Osler Health Center, Toronto, Ontario, Canada; 13 Renopath, Center for Renal and Urological Pathology, Chennai, India; 14 Renal Pathology and Electron Microscopy, Dr Lal PathLabs, New Delhi, India; 15 Aster Mims Kozhikode, India

**Keywords:** cyclophosphamide, membranous nephropathy, NELL1, PLA2R, rituximab, steroids

## Introduction

Among patients with primary membranous nephropathy (PMN), neural epidermal growth factor-like 1 protein (NELL1)-associated membranous nephropathy (MN) is the second most common antigen, following phospholipase A2 receptor (PLA2R).^1^

The natural history of PLA2R-associated MN is well described. However, the natural history of other antigen-associated MN is incompletely understood. Emerging evidence suggests that complementary and alternative medicine (CAM) and malignancies may play a role in the development of NELL1-associated MN.^2^, ^3^, ^4^ According to the study by Caza and colleagues, only a quarter of patients with NELL1-associated MN received immunosuppression, signaling that immunosuppression may not have a significant impact on the clinical outcomes.^3^ Despite a growing understanding of the underlying etiology of NELL1-associated MN, more studies are needed to determine treatment practices and clinical outcomes. In addition, it is difficult to discern whether NELL1-associated MNs are associated with secondary causes differently from unidentified antigen-associated MNs. Considering the aforementioned knowledge gap, this study examined the clinical characteristics and remission rates of NELL1-associated MNs and compared them with unidentified antigen-associated MNs whose clinical characteristics and therapeutic outcomes remain unclear. The methods are described in the Supplementary Methods.

## Results

The study included 46 and 36 patients with NELL1-associated and unidentified antigen-associated MN, respectively. The mean age of the patients in the NELL1 and unidentified antigen-associated MN group was 40.7 ± 15.3 (18–75) and 40.3 ± 13.6 (16–68) years, respectively. The pooled incidence of NELL1-associated PMN was 11.5%, and 58.6% of patients had a history of CAM exposure, compared to 16.7% in the unidentified antigen group (*P* < 0.0001). Baseline parameters and outcomes are shown in Table 1.

**Table 1:** Baseline and outcome parameters

Variables	Unidentified antigen-associated MN (*n* = 36)	NELL1 group (*n* = 46)
Baseline characteristics		
Mean age (yr)	40.3 ± 13.6 (16–68)	40.7 ± 15.3 (18–75)
M/F (*n*)	20/16	22/24
CAM, *n* (%)	6 (16.7)^a^	27 (58.6)^a^
Hypertension, *n* (%)	18 (50.0)	5 (10.8)
Diabetes mellitus, *n* (%)	1 (2.7)	7 (15.2)
Duration (mo)	6 (1,12)	6 (4,6.7)
Proteinuria (g/d) (median)	5.9 (3.5–9.6)	7.7 (4.5–11.7)
Serum albumin (g/dl) (mean)	2.4 ± 0.7 (1.1–3.9)	2.5 ± 0.7 (1.2–4.2)
Serum creatinine(mg/dl) (median)	0.9 (0.6–1.2)	0.8 (0.6–1.0)
Treatment and outcome, *n* (%)		
Conservative management	4 (11.1)	20 (43.4)
Immunosuppression	32 (88.9)^b^	26 (56.5)^b^
1. Rituximab	7 (21.9)	8 (30.7)
2. Cyclical CYC/CS	23 (71.9)	18 (69.2)
3. Others	2 (6.2)	None
Treatment response		
1. Clinical remission	24 (68.5)	33 (76.7)
2. Complete remission	12 (50.0)	11 (33.3)
3. Partial remission	12 (50.0)	22 (66.7)
Number of deaths (*n*)	1	1
Lost to follow-up (n)	1	3
Follow-up^c^ (median duration) (mo)	24 (12,36)	10 (6,18)
Proteinuria (g/d)	0.9 (0.2–2.8)	1.6 (0.4–3.2)
Serum albumin (g/dl)	3.7 ± 0.8	3.7 ± 0.6
Serum creatinine (mg/dl)	0.8 (0.6–1.8)	0.8 (0.6–1)

CAM, complementary and alternative medicines; CYC/CS, cyclophosphamide-steroids; F, female; M, male; MN, membranous nephropathy; NELL1, Neural epidermal growth factor-like 1 protein.

a *P* = 0.0001.

b *P* = 0.001.

c During the follow-up, 2 patients died and 4 were lost to follow-up.

### Pathology

Segmental IgG staining was more prevalent in NELL1-associated MN (47.8%) than in the unidentified antigen-associated MN (3.22%). C3 staining was positive in three-fourths of cases (73.9% in the NELL1-related MN and 69.4% in the unidentified antigen-associated MN) (Supplementary Table S1).

### Clinical Outcomes

Compared to the NELL1 group (56.5%), the prescription of immunosuppressive therapy was higher in the unidentified antigen group (88.9%) (*P* = 0.001). Thirty-three (76.7%) and 24 (66.6%) patients in the NELL1-associated and unidentified antigen-associated MN achieved remission, respectively (Table 1, Supplementary Figure S1). As a result of the withdrawal of the inciting agent, 9 patients (33.3%) in the NELL1 group and 1 patient (16.66%) in the unidentified antigen group achieved clinical remission (Supplementary Figure S1). Among the patients with NELL1-associated MN, 13 of 20 (65%) achieved remission with supportive therapy (spontaneous remission, with CAM intake: 9 of 14 [64.2%] and with no CAM intake: 4 of 6 [67%]) as compared to 2 (50%) in the unidentified antigen group. With immunosuppressive therapy, 21 patients (80.7%) in the NELL1 group and 22 patients (68.75%) in the unidentified antigen-associated MN group achieved clinical remission (Supplementary Figure S1). One patient with NELL1-associated MN relapsed during follow-up and had remission following the withdrawal of skin-whitening cream. One patient with HIV-positive and NELL1 MN was on Highly active antiretroviral therapy for 7 years (undetectable viral load and a CD4 count of 555 cells/ml). The patient was referred to the nephrology unit due to pedal oedema and proteinuria. Following cyclical cyclophosphamide and steroid therapy,S1 the patient has now achieved remission.

## Discussion

Our study examined the clinical, biochemical, therapeutic, and outcome characteristics of NELL1-associated and unidentified antigen-associated MN patients. Approximately half of the patients in the NELL1 group had a history of consuming CAM. Immunosuppressive therapy was required far less frequently in the NELL1 group than in the group with unidentified antigens.

A variety of clinical settings have been linked to NELL1-associated PMN, which includes cancer, drugs, infections, autoimmune diseases, stem cell transplantation, and *de novo* MN following kidney transplantation.^3^^,^^5^ An analysis of 126 patients by Sethi *et al.*^6^ revealed that 23% of the 126 PLA2R-negative cases were NELL1-positive. Nonetheless, in 1 series from China, a third of the PLA2R-negative and THSD7A-negative PMNs were NELL1-related; however, none displayed any signs of malignancy.^7^ Although all patients in the present study underwent a reasonable malignancy screening, none showed any signs of cancer. In an interesting finding, CAM consumption was associated with both PLA2R-related^8^ and NELL1-related MN.^2^^,^^4^ Kurien *et al.*^2^ reported that approximately 35% of MN cases in India were associated with traditional indigenous medicine use, of which 88% were NELL1-positive. A meticulous history is essential; in the present study, a small minority consumed CAM after the onset of their illness, which may be an incorrect attribution to PMN development. Emerging evidence suggests that mercury and lipoic acid-containing CAM are associated with developing NELL1-associated PMNs.^4^^,^^9^ The comprehensive summary of NELL1 case reports-based reviewS2–S9 is shown in Supplementary Table S2.

As reported by Sethi *et al.*^6^ 16% of their patients with PMN had granular staining with NELL1 antibody, which was similar in pattern to IgG. In these cases, there was incomplete or segmental staining with IgG, prominent IgG1, mild-to-moderate staining with C3, increased mesangial deposits, and reduced extraglomerular staining. In the current study, segmental IgG staining was observed in 50% of the NELL1-associated MN cases. Similarly, C3 staining was observed in three-fourths of the NELL1 group and the unidentified antigen group. The results of the present study are similar to the finding of Caza *et al.*,^3^ who found pure segmental staining (IgG) in 45% of the cases and C3 staining in three-fourths of the cases.

Currently, very little information is available regarding the clinical outcomes in patients with (non–malignancy-related) NELL1-associated and unidentified antigen-associated PMN. In the study by *Caza et al.*,^3^ only 15 patients (25.42%) received immunosuppressive therapy. A total of 8 patients were treated with calcineurin inhibitors, 3 with cyclophosphamide, 3 more with mycophenolate mofetil combined with steroids, and 1 with rituximab therapy. The granular details of the cases in the aforementioned series is unavailable. Sultan *et al.*^9^ reported a successful response to cyclical cyclophosphamide-corticosteroids in 2 patients with skin-whitening cream-associated NELL1 MN. In the present study, immunosuppression was less frequently required in the NELL1 group than in the unidentified antigen group, perhaps partly attributed to the higher rate of CAM intake in the NELL1 group, which may have responded swiftly to the withdrawal of the inciting agent. Despite the differences in therapeutic approach in both groups, clinical remission rates were comparable. A descriptive study by Wang *et al.*^7^ reported follow-up data for 12 patients, of whom 10 received immunosuppressive therapy, and 11 achieved clinical remission (complete remission: 5 and partial remission: 6). The predominant immunosuppressive agents used in our study were cyclical cyclophosphamide-corticosteroid or rituximab only therapy. In both the NELL1 and unidentified antigen groups, there was no difference in remission rates between patients treated with cyclical cyclophosphamide-corticosteroid and those treated with rituximab. In the NELL1 group, all but 1 patient treated with rituximab experienced clinical remission. Although constrained by a small sample size, the present study suggests that rituximab could prove efficacious in managing anti-proteinuric resistant NELL1-related PMN (Supplementary Figure S2).

### Conclusion

Although this is the first study to extensively delineate the clinical outcomes of NELL1-associated PMN and juxtapose them with those of unidentified antigen-associated PMN, this study is not without its limitations. These encompass a modest sample size, brief follow-up duration, a dearth of information regarding the nature of CAM, absence of antibody monitoring, lack of IgG-subtyping, and a nonprotocolized approach to management. In conclusion, NELL1-associated PMN demonstrates an association with CAM intake compared to the unidentified PMN group. Two-thirds of patients in both cohorts achieved clinical remission, with spontaneous remission being more prevalent in the NELL1 group.

## Disclosure

RR received scientific grants from ICMR and PGIMER intramural grants. All the other authors declared no competing interests.

## Supplementary Material

**Supplementary Methods.**

**Supplementary References.**

**Figure S1.** Clinical characteristics and outcomes.

**Figure S2.** Approach to manage NELL1 associated membranous nephropathy.

NELL1 case reports-based review.

**Table S1.** Biopsy details.

**Table S2.** Summary of case reports of NELL1-associated membranous nephropathy.

## References

[1] Ronco P., Beck L., Debiec H. (2021). Membranous nephropathy. Nat Rev Dis Primers.

[2] Kurien A.A., Ks P., Walker P.D., Caza T.N.J., walker P.D., Caza T.N. (2022). Traditional indigenous medicines are an etiologic consideration for NELL1-positive membranous nephropathy. Kidney Int.

[3] Caza T.N., Hassen S.I., Dvanajscak Z. (2021). NELL1 is a target antigen in malignancy-associated membranous nephropathy. Kidney Int.

[4] Spain R.I., Andeen N.K., Gibson P.C. (2021). Lipoic acid supplementation associated with neural epidermal growth factor-like 1 (NELL1)–associated membranous nephropathy. Kidney Int.

[5] Münch J., Krüger B.M., Weimann A. (2021). Posttransplant nephrotic syndrome resulting from NELL1-positive membranous nephropathy. Am J Transplant.

[6] Sethi S., Debiec H., Madden B. (2020). Neural epidermal growth factor-like 1 protein (NELL-1) associated membranous nephropathy. Kidney Int.

[7] Wang G., Sun L., Dong H. (2021). Neural epidermal growth factor–like 1 protein–positive membranous nephropathy in Chinese patients. Clin J Am Soc Nephrol.

[8] Kaur P., Prabhahar A., Kumar A. (2023). Complementary medicine and phospholipase A2 receptor (PLA2R)–related membranous nephropathy—fortuitous or causal?. Kidney Int.

[9] Sultan A., Mamankar D., Thakare S. (2023). Mercury-associated neural epidermal growth factor-like 1 protein (NELL-1) positive membranous nephropathy after use of skin lightening creams. Clin Toxicol (Phila).

