# Supplementary material for: Clinical Characteristics and Outcomes of Neural Epidermal Growth Factor-like 1 Protein-Associated Membranous Nephropathy
Source: Kidney Int Rep. 2024 Feb 24;9(5):1513–6. doi: 10.1016/j.ekir.2024.02.1405 (PMC11068952; doi:10.1016/j.ekir.2024.02.1405)
Supplement: Supplementary File (PDF) [file mmc1.pdf]

Supplementary methods

Study design and setting

This retrospective descriptive study describes patients with NELL1-associated membranous nephropathy from various centres (Post Graduate Institute of Medical Education and Research, Chandigarh, LTMC, Sion, Mumbai, IQRAA International Hospital and Research, Centre Kozhikode, All India Institute of Medical Sciences, Bathinda, Dr. Hedgewar Hospital, Aurangabad, Madras Medical Mission, Chennai, Lifeline Hospital, Thane, Max Superspeciality Hospital, Saket, New Delhi, Bombay Hospital, and Medical Research Center Mumbai and Vardhman Mahavir Medical College, Delhi) in India and compares their baseline parameters and clinical outcomes with unidentified antigen-associated membranous nephropathy.

Participants

The study included consecutive patients (age≥16 years) with NELL1-associated membranous nephropathy from various Indian centres and patients with unidentified antigen-associated membranous nephropathy from the PGIMER PMN registry. The study excluded patients with lupus nephritis or other connective tissue disorders.

Protocol and data collection

Both groups were profiled in terms of their demographic parameters, clinical, biochemical, and histological characteristics. Immunosuppression was administered at the discretion of the treating physician. We recorded the baseline and last serum albumin, creatinine, and proteinuria. All the patients underwent active screening for cancers.

Outcomes and definitions

Nephrotic syndrome was defined as proteinuria of ≥3.5 gm/day or ≥2 gm/day, along with serum albumin of <2.5 gm/dl or ≥2 gm/day, along with serum albumin of <2.5 gm/dl (1). Remission was classified as complete remission (CR): Proteinuria of ≤ 0.3 g/day with normal serum albumin and stable serum creatinine or partial remission (PR): Proteinuria > 0.3 gm/day but < 3.5 gm/day and > 50% of baseline with normal serum albumin (≥ 3.0 gm/dl) and stable serum creatinine. Resistant: Patients who failed to achieve CR, PR, developed end-stage kidney disease (ESKD) or died (irrespective of remission status) or required additional immunosuppressive therapy to achieve remission. Unidentified antigen-associated MN: Patients with biopsy-proven MN and immunohistochemistry (IHC) of the kidney biopsy negative for PLA2R, NELL1, THSD7A, Semaphorin, and Exostosin-1/2. NELL1 associated MN: Patients with biopsy-proven MN with NELL1 positive by IHC and negative for PLA2R.

Statistical methods

The data is presented as numbers, percentages, the mean and standard deviation (range) for normally distributed variables, and median and interquartile range when the variables are not normally distributed. Chi-square tests, student T-tests, and Mann-Whitney tests were used to compare the parameters between the two groups. The statistical analysis was performed using GraphPad Prism (Version 10.0, La Jolla, California, United States). Statistical significance was defined as a p-value of less than 0.05.

Supplemental Table S1: Biopsy details

S no	Parameters	NELL1-related (n=46)	Unidentified Ag-related (n=36)
**1**	GBM thickening	**34/46 (73.9%)**	**36/36 (100%)**
**2**	IgG staining	**46/46 (100%)**	**36/36 (100%)**
**3**	C3 staining	**34/46 (73.9%)**	**25/36 (69.4%)**
**4**	Segmental staining	**22/46 (47.8%)**	**5/36 (13.8%)**
**5**	Chronicity (IFTA>30%)	**07/46(15.2%)**	**04/36 (11.1%)**

Ag- antigen, GBM- glomerular basement membrane, IgG- immunoglobulin G and C- complement

Supplemental Figure S1: Clinical characteristics and outcomes

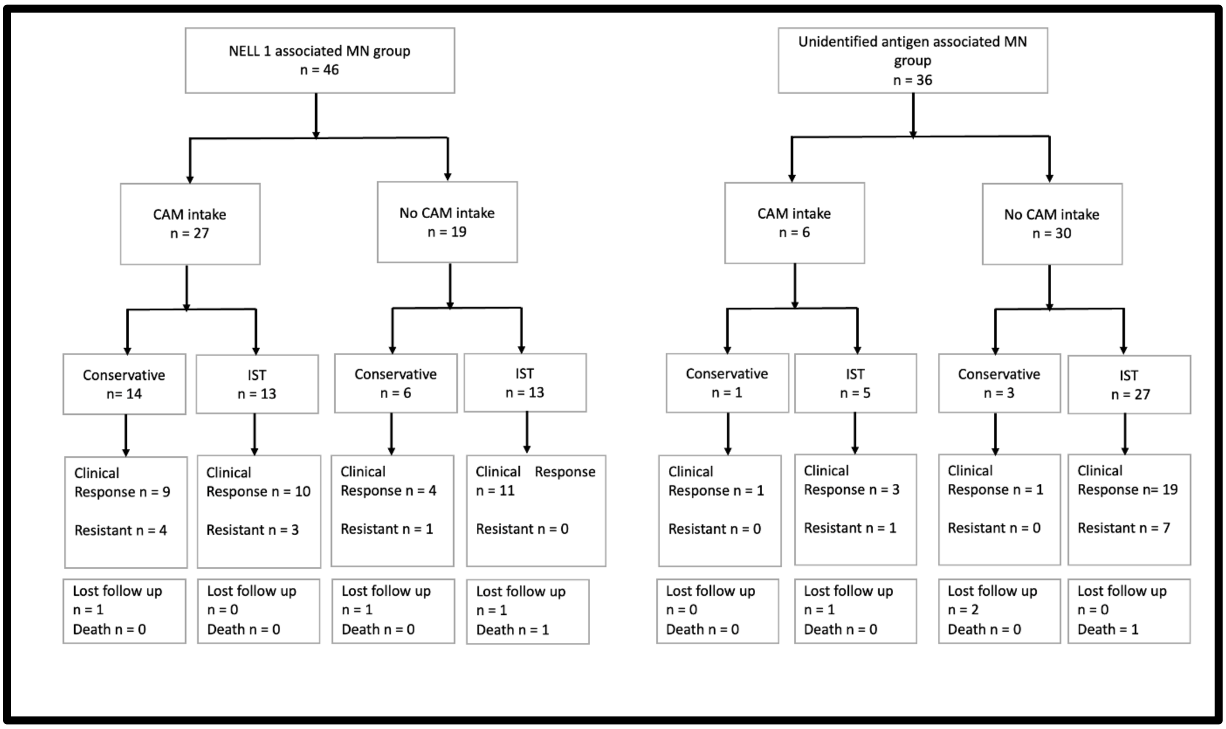

Footnote: NELL-1 - Neural epidermal growth factor-like 1 protein, MN – Membranous nephropathy, CAM – complementary and alternative medicines, IST- immunosuppressive therapy

Supplemental Figure S2: Approach to manage NELL1 associated MN

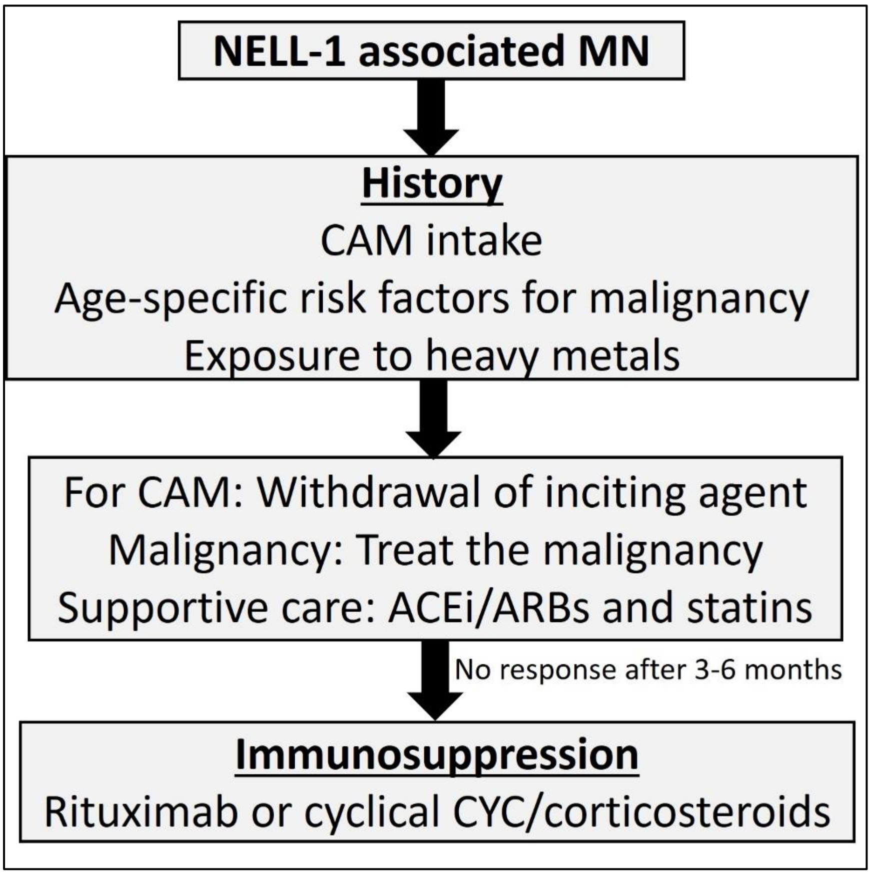

Footnote: NELL-1 - Neural epidermal growth factor-like 1 protein, CAM – complementary and alternative medicines, ACEi – Angiotensin-converting enzyme inhibitors, ARB-Angiotensin receptor blocker, CYC - Cyclophosphamide

NELL1 case reports-based review

A systematic PubMed search using the keyword "NELL1-associated membranous nephropathy" from 2020 to 2023 yielded 14 case reports of NELL1-associated membranous nephropathy (NELL1-MN). These cases exhibited a wide range of clinico-demographic features, with a mean age of 53.35 ± 18.83 years and a notable female predominance (71.42%). Nephrotic syndrome was the most common presentation, with a median proteinuria and serum albumin of 5.05 g/day and 2.39 g/dL, respectively. Ten patients (71.42%) had a prior history of complementary and alternative medicine (CAM) use, of which four (28.57%) cases had a history of lipoic acid supplementation, and two (14.28 %) cases had raised whole blood mercury levels. Segmental IgG staining was evident in half of the patients. Four patients received immunosuppressive therapy (28.57%), of which two received cyclical cyclophosphamide and steroids. Encouragingly, 85.7% (n=12) of patients responded to treatment, with complete and partial remission in 50% (n=7) and 35.7% (n=5) of cases, respectively, while one case remained resistant.

Supplemental Table S2: Summary of case reports of NELL1-associated MN

Author	Age	Proteinuria (g/d)	S.albumin (g/dl)	S.Creatinine (mg/dl)	Associated findings	Renal biopsy	IS	Remission (CR/PR)
**Pathak et al.2022(2)**	47/F	10	2.1	0.4	• Neck swelling -Lymphocytic thyroiditis<br>• Elevated urine mercury<br>• CAM medication intake	Global capillary wall staining for IgG , C3 ,C1q,Kappa, Lambda ?nell	No	PR
**Dinesh et al.2022(3)**	65/M	9.0	2.8	1.0	• HIV-1 RNA PCR <20/µL<br>• Absolute CD4 count was 341 cells/µL	Full house pattern?NELL1	No	CR
**Munch et al.2021(4)**	56/M	4.0	2.3	1.52	• Post renal transplant<br>• ALS on Riluzole	IgG4 staining with NELL1 positivity	Continued with the triple IS	PR
**Rebecca et al.2021(5)**	56/F	19	N/A	0.6	Lipoic acid supplementation	IgG1 segmental staining with NELL1 positivity	No	CR
	74/F	2.1	N/A	0.6	• Lipoic acid supplementation<br>• Basal cell carcinoma	IgG segmental staining with NELL1 positivity	No	CR
	66/F	4.4	N/A	0.7	• Lipoic acid supplementation<br>• Multiple sclerosis	Biopsy not done	No	CR
	41/F	5.5	N/A	0.74	• Lipoic acid supplementation<br>• Celiac disease	IgG segmental staining with NELL1 positivity	No	PR
	71/F	4.5	N/A	0.67	• Lipoic acid supplementation<br>• Smoldering multiple myeloma	IgG1 segmental staining with NELL1 positivity	No	PR
**Santoriello et al.2023(6)**	53/M	4.6	4.5	1.1	• Type2 DM<br>• Cystinuria<br>• Tiopronin intake	IgG segmental staining with NELL1 positivity	No (withdrawal of Tiopronin)	PR
**Inoue et al. 2023(7)**	70/M	6.2	3.2	0.86	• Serum anti PLA2R antibody was positive	IgG1 segmental staining with NELL1 positivity	Yes (steroids)	CR
**Sultan et al. 2023(8)**	17/F	6.4	2.4	0.63	Elevated whole blood mercury levels 46.24 mg/L (0.23 mmol/L)	IgG4 and IgG1 global staining with NELL1 positivity	Yes(steroid/CYC)	Resistant
	39/F	10.2	1.85	1	Elevated whole blood mercury levels 57.08 mg/L (0.29 mmol/L)	IgG1 ,IgG4 and IgG2 global staining with NELL1 positivity	Yes(steroid/CYC)	CR
	19/F	2.0	2.38	1.07	N/A	N/A	No	CR
**Jiffry et al.2023(9)**	73/F	2.1	N/A	5.28	Type 2 DM and Heart failure with preserved EF	IgG segmental staining with NELL1 positivity	No	N/A

NELL-1 - Neural epidermal growth factor-like 1 protein , MN – membranous nephropathy, IS – immunosuppression, PR/CR – partial remission/complete remission, N/A – not available, EF – ejection fraction, DM- Diabetes mellitus , triple immunosuppression – Tacrolimus, Mycophenolate mofetil and steroids, S alb – serum albumin , S creat – serum creatinine , steroid/CYC -cyclical steroid and cyclophosphamide ,PLA2R – Phospholipase A2 receptor

Supplementary References

S1. Ramachandran R, Hn HK, Kumar V, Nada R, Yadav AK, Goyal A, et al. Tacrolimus combined with corticosteroids versus Modified Ponticelli regimen in treatment of idiopathic membranous nephropathy: Randomized control trial. Nephrology. 2016 Feb;21(2):139–46.

S2. Pathak N, Gunasekaran I, Ambriose M, Nanda S. Nell1 as Target Antigen for Mercury Related Membranous Nephropathy: A Case Report. Indian J Nephrol. 2022;32(5):502.

S3. Dinesh KP, Charu V, Troxell ML, Andeen NK. NELL1-Positive HIV-Associated Lupus-Like Membranous Nephropathy with Spontaneous Remission. Glomerular Dis. 2022 Jun 29;2(4):184–8.

S4. Münch J, Krüger BM, Weimann A, Wiech T, Reinhard L, Hoxha E, et al. Posttransplant nephrotic syndrome resulting from NELL1-positive membranous nephropathy. American Journal of Transplantation. 2021 Sep;21(9):3175–9.

S5. Spain RI, Andeen NK, Gibson PC, Samuels MH, Morris CD, Solomon AJ, et al. Lipoic acid supplementation associated with neural epidermal growth factor-like 1 (NELL1)–associated membranous nephropathy. Kidney International. 2021 Dec;100(6):1208–13.

S6. Santoriello D, Ramaswamy R, Kudose S, Markowitz GS. Segmental NELL-1 Membranous Nephropathy Complicating Tiopronin Therapy. Kidney International Reports. 2023 Aug;8(8):1683–6.

S7. Inoue D, Uchida T, Komatsu S, Sugisaki K, Yamada M, Ogawa H, et al. Anti-PLA2R Antibody Development During NELL1-Associated Membranous Glomerulonephritis Treatment: A Case Report. Kidney Medicine. 2023 May;5(5):100625.

S8. Sultan A, Mamankar D, Thakare S, Rojekar A, Jamale T. Mercury-associated neural epidermal growth factor-like 1 protein (NELL-1) positive membranous nephropathy after use of skin lightening creams. Clinical Toxicology. 2023 May 4;61(5):387–91.

S9. Mohamed Jiffry MZ, Pitts K, Munir M, Khan A, Josephs M. A Rare Case of Neural Epidermal Growth Factor-Like 1 Protein (NELL-1) Antigen-Associated Membranous Nephropathy. Cureus [Internet]. 2023 Feb 7 [cited 2023 Jun 26]; Available from: https://www.cureus.com/articles/136994-a-rare-case-of-neural-epidermal-growth-factor-like-1-protein-nell-1-antigen-associated-membranous-nephropathy
